# Prevalence, distribution and antimicrobial susceptibility pattern of bacterial isolates from a tertiary Hospital in Malawi

**DOI:** 10.1186/s12879-020-05725-w

**Published:** 2021-01-07

**Authors:** Pizga Kumwenda, Emmanuel C. Adukwu, Ebot S. Tabe, Victor. C. Ujor, Pocha S. Kamudumuli, Maono Ngwira, Joseph Tsung Shu Wu, Master R. O. Chisale

**Affiliations:** 1 Department of Biomedical Sciences, Faculty of Health Sciences, Mzuzu University, Private Bag 201, Luwinga, Mzuzu 2, Malawi; 2 Centre for Research in Biosciences, Faculty of Health and Applied Sciences, University of the West of England, Bristol, BS16 1QY UK; 3 Albany College of Pharmacy and Health Sciences, Albany, NY 12208 USA; 4 Bioenergy and Water Treatment Management Program, Agricultural Technical Institute, The Ohio State University, 1328 Dover Road, Wooster, OH 44691 USA; 5 Maryland Global Initiative Corporation, Golden Peacock Shopping Complex, Area 13, City Centre, P.O Box 2298, Lilongwe, Malawi; 6 Luke International (LIN), Box 1088, Mzuzu, Malawi; 7 Pingtung Christian Hospital (PTCH), Kaohsiung, Taiwan; 8 Department of Biological Sciences, Faculty of Science, Technology and Innovation, Mzuzu University, Private Bag 201, Luwinga, Mzuzu 2, Malawi

**Keywords:** Antimicrobial resistance, Antibiotics, Prevalence, Susceptibility, Malawi

## Abstract

**Background:**

Bacterial infections are a significant cause of sickness and death in sub-Saharan Africa. This study aimed at establishing the prevalence, distribution and antimicrobial susceptibility pattern of major bacterial isolates from patients accessing medical care at a tertiary hospital in Malawi.

**Methods:**

We retrospectively reviewed bacteria culture and antimicrobial susceptibility records for 4617 patients from 2002 to 2014 at Mzuzu Central Hospital (MCH). No inclusion and exclusion criteria were followed. Data was analysed using excel (Microsoft office, USA) and GraphPad prism 7 software programs.

**Results:**

The most prevalent isolates were *S. aureus* (34.7%, *n* = 783), *Klebsiella* species (17.4%, *n* = 393) and *Proteus* species (11.4%, *n* = 256). Most microorganisms were isolated from adults (88.3%, *n* = 3889) and pus was the main source (69.3%, *n* = 1224). *S. pneumoniae* was predominantly isolated from cerebrospinal fluid (60.3%, *n* = 44) largely collected from children (88.2%, *n* = 64). Overall, most bacteria exhibited high resistance to all regularly used antimicrobials excluding ciprofloxacin.

**Conclusions:**

Our report demonstrates an increase in bacterial infection burden in sites other than blood stream and subsequent increase in prevalence of antimicrobial resistance for all major isolates. Creating an epidemiological survey unit at MCH will be essential to help inform better treatment and management options for patients with bacterial infections.

## Background

Bacterial diseases remain the major cause of morbidity and mortality worldwide [1]. Infectious diseases are predominant in Africa and are largely influenced by vulnerable population, social inequality and poor health delivery systems [1]. Inadequate funding, poor microbiology services, limited technical experts and scarce epidemiological data to inform better preventive and treatment strategies all contribute to the rise in burden of bacterial infections in Africa [2, 3]. This increase in the burden of bacterial infections is also accompanied by high incidences of antimicrobial resistance (AMR) [4]. As a low income country in Africa, Malawi is no exception to these challenges [5].

Across Africa, AMR has been reported in different countries. Apart from the emergence of Methicillin-resistant *Staphylococcus aureus* (MRSA), Malawi has reported a rapid increase in *non-Salmonella* Enterobacteriaceae with extended spectrum beta-lactamase (ESBL) and fluoroquinolone resistance among common Gram-negative pathogens [5]. In Zimbabwe, Gram-negative bacteria showed high resistance to cotrimoxazole (68.5%) and ampicillin (84.5%) while Gram-positive bacteria demonstrated resistance to cotrimoxazole (69%) and Nalidixic acid (81%) [6]. A study in Ghana revealed that *Neisseria gonorrhoeae* isolates were resistant to tetracycline (100%), benzylpenicillin (90.9%) and ciprofloxacin (88.6%) [7]. In general, important Gram-negative bacteria including *Klesbsiella*, *E. coli*, *Salmonella*, *Shigella* and Gram-positive bacteria such as *S. pneumoniae, S. aureus, S. agalactae, (MRSA*) across Africa have acquired resistance to the common essential antimicrobials being used in the region [8].

The rise in AMR has been attributed to several factors such as bad clinical practices, poor public perception and behaviour towards antimicrobial use, increase in adverse agricultural practices etc. [9]. Most importantly, irresponsible widespread use of antimicrobials creates a sturdy selective pressure that propels the adaptive and evolutionary response by microbes, hence the emergence of various AMR phenotypes [9]. Worse still, today’s global economy allows people to travel and interact worldwide which enables transmission and spread of pathogens worldwide [9]. The rise in AMR not only threatens the public health sector but also the already fragile economies in the African continent through prolonged hospitalization and use of expensive and specialised treatment and care for the sick.

It is generally accepted that AMR is a global threat mostly affecting low-income countries, however most data available on bacterial pathogen surveillance and AMR profile is from high-income countries [8]. Nevertheless, in Africa, many countries use syndromic approaches to treat bacterial infections. In order to be effective, empirical treatment requires better knowledge of local AMR profiles. The aim of this study was to determine the prevalence, distribution and antimicrobial susceptibility patterns of commonly isolated pathogenic bacteria at Mzuzu Central Hospital (MCH), a referral hospital for six district hospitals in the Northern region of Malawi.

## Methodology

### Research design

We conducted a retrospective audit of records for bacterial culture and susceptibility testing results between the years 2002 and 2014 from MCH microbiology laboratory department. No inclusion and exclusion criteria were applied. The MCH Laboratory participates in international microbiology quality assessment programs such as the UK External Quality Assessment program (UKNEQAS) and Zimbabwe Quality Assurance Program (ZIMQAP). Ethical approval for the study was obtained from National Health Science Research Committee (NHSRC; approval number NHSRC # 1206).

### Bacterial identification and characterisation

Specimens from outpatient department and clinical wards were transported to microbiology department laboratory for analysis within 2 h of collection. Conventional microbiological culture methods were employed to isolate and identify bacteria. Media was prepared in-house as per procedures stipulated in Cheesbrough [10].

About 10 mL and 3 mL of blood collected from adults and paediatric patients, respectively were inoculated into BD Bactec plus aerobic culture vials™ for incubation at 35–37 °C in BD Bactec™ 9050 Blood Culture System (BD, USA). Positive cultures were sub-cultured onto Blood Agar Plate (BAP), MacConkey Agar Plate (MAP) or Chocolate Agar Plate (CAP) if any fastidious organism was suspected. Inoculated plates were incubated at 37 °C, 5% CO_2_ for up to 72 h.

Swabs collected from wounds, skin and soft tissue infection were Gram stained, cultured on either BAP/CAP or MAP, and, incubated at 37 °C,5% CO_2_ for up to 72 h [10]. Swabs collected from cerebrospinal fluid (CSF) and other fluids, were Gram stained, cultured on BAP and CAP for incubation at 37 °C,5% CO_2_ for up to 72 h [10]. Urine samples were inoculated on CLED agar and incubated at 37 °C for up to 48 h [10]. A specimen was considered positive for urinary tract infection (UTI) if the number of enumerated colonies were greater than ≥10^5^ CFU/mL [10, 11].

Isolates were identified and processed according to standard techniques summarised in Table 1. Antibiotic susceptibility was determined by disc diffusion technique on Mueller -Hinton agar except for *Streptococcus pneumonia* and other fastidious organisms, the Mueller-Hinton agar was supplemented with 5% human blood (from blood donors). Data was interpreted according to the CLSI guidelines with quality-controlled strains; *Escherichia coli* ATCC 25922 and *Staphylococcus aureus* ATCC 25923 [12]. Major bacterial isolates were evaluated for their susceptibility against the most commonly used antimicrobials during the study period. Antimicrobial screening was performed with the following antibiotics: ciprofloxacin, ceftriaxone, penicillin, tetracycline, ampicillin, chloramphenicol, amoxicillin, and gentamicin.

**Table 1:** Laboratory methods for bacteria identification and susceptibility testing

Organism	Media	Tests for presumptive identification	Susceptibility testing Method
*Enterobacteriaceae* and other Gram-negative bacilli	BAP MAP	Gram stain Colony characteristics Indole test Methyl red test Voges-Proskauer test Citrate utilization test Urease test Triple sugar iron (TSI) test	Disc diffusion
*Haemophilus* species	BAP MAP	Gram stain Colony characteristics Hemolysis X and V growth factor requirements	Disc diffusion
*Neisseria* species	BAP CAP TMA	Gram stain Colony characteristics Catalase test Oxidase test Growth on Thayer-Martin	Disc diffusion
*Streptococci* species	BAP CAP	Gram stain Hemolysis Bile solubility test Optochin susceptibility Bacitracin susceptibility	Disc diffusion
*Staphylococci* species	BAP CAP MSA	Gram stain Catalase test Coagulase test Novobiocin susceptibility	Disc diffusion
*Pseudomonas* species	BAP MAP	Gram stain Colony characteristics Oxidase test Oxidative fermentation	Disc diffusion

### Data analysis

Data was analysed using Excel (Microsoft office, USA) and GraphPad Prism 7 software (San Diego, CA, USA). Graphs or pie chart were used to show the prevalence and distribution of the isolated bacteria against gender (male and female), age groups (Children and adults) and specimens (Pus, ear swab, V. Blood, Cerebral Spinal Fluids, High Vaginal Swabs, Urine and Other fluids). In addition, a frequency table expressed in percentage and absolute numbers was used to display the susceptibility patterns of the six commonly isolated bacteria against the six commonly used antibiotics.

## Results

### Study population and demographics

**Fig. 1:**
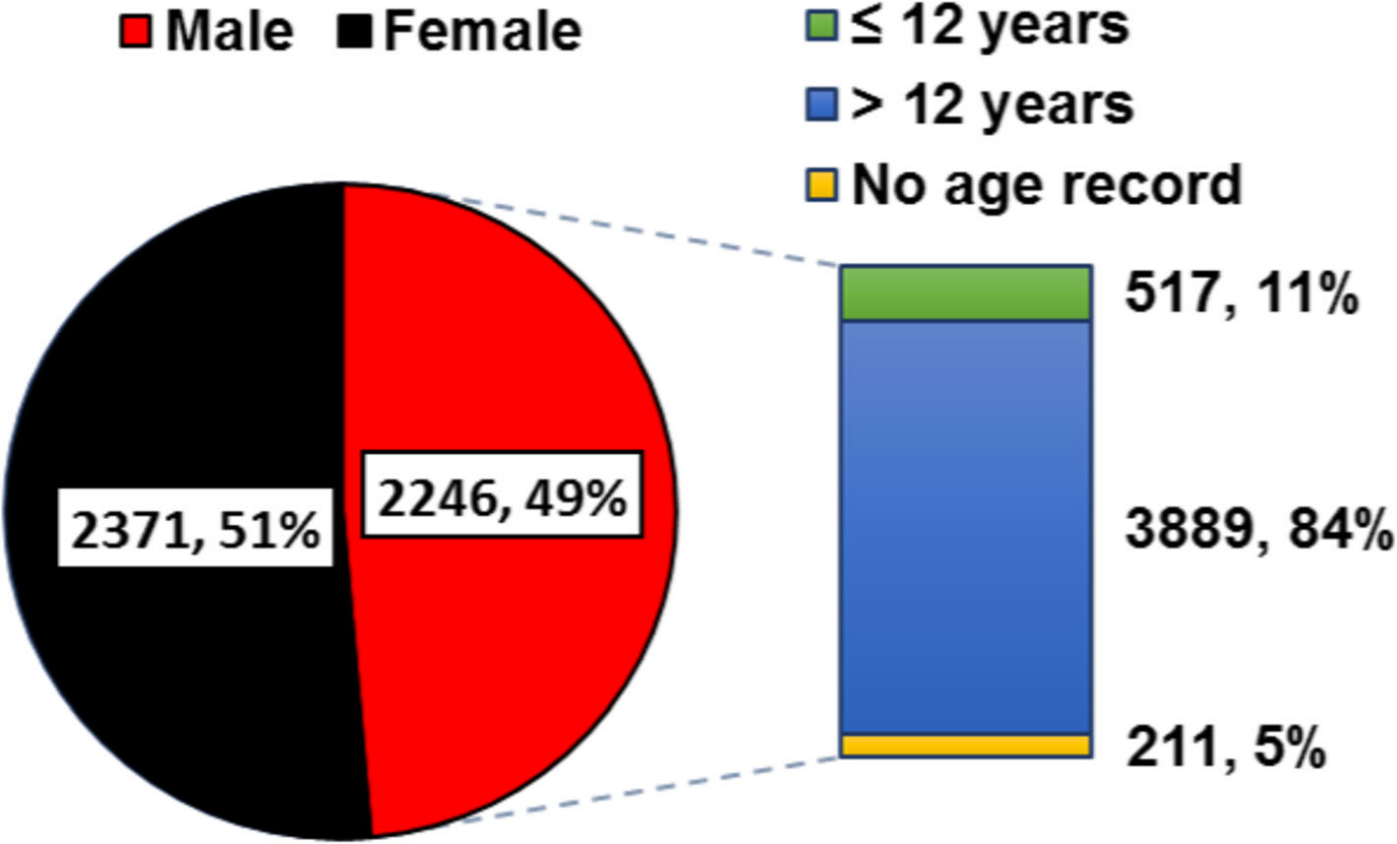
Demographics of the study population. Patients were categorised by gender and age group

To understand the demographics of the patient population, clients were characterised according to their sex and age group. Out of 4617 patients who submitted samples for microbial analysis, 2246 (49%) were male and 2371 (51%) were female (Fig. 1). We observed that 84% (*n* =3889) were adult patients (> 12 years old) while were 11% (*n* =517) were children (≤12 years old) (Fig. 1).

**Fig. 2:**
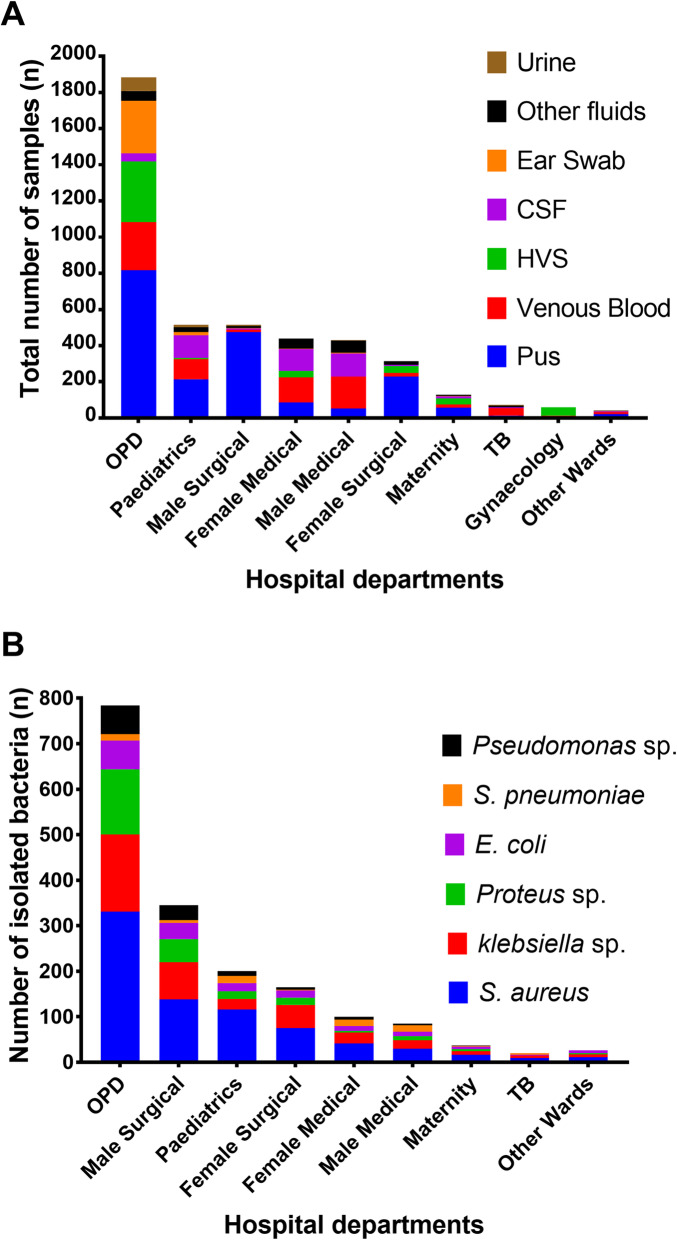
Distribution of isolates by specimen source and hospital department. Absolute number of (**a**) specimens and (**b**) isolates recovered per department were determined

### Analysis of isolates by source of specimen and hospital department

To analyse the dynamics of bacterial infections, total number of specimens collected and isolates recovered were determined for each hospital department. Most samples were collected in the Out Patient Department (OPD;1885, 43%; Fig. 2a). Pus from wound and ear infections were the most frequent samples and the majority of them were collected in the OPD, male surgical ward, paediatric ward and female surgical ward (Fig. 2a). Corresponding to the high number of samples collected, the OPD had the highest number of bacterial isolates (784, 44.5%; Fig. 2a). TB ward had the least number of isolates (20, 1.1%). Across the departments, *S. aureus* was the most isolated pathogen (783, 43.5%) followed by *Klebsiella* species (387, 22%) (Fig. 2b).

### Prevalence of bacterial isolates

**Fig. 3:**
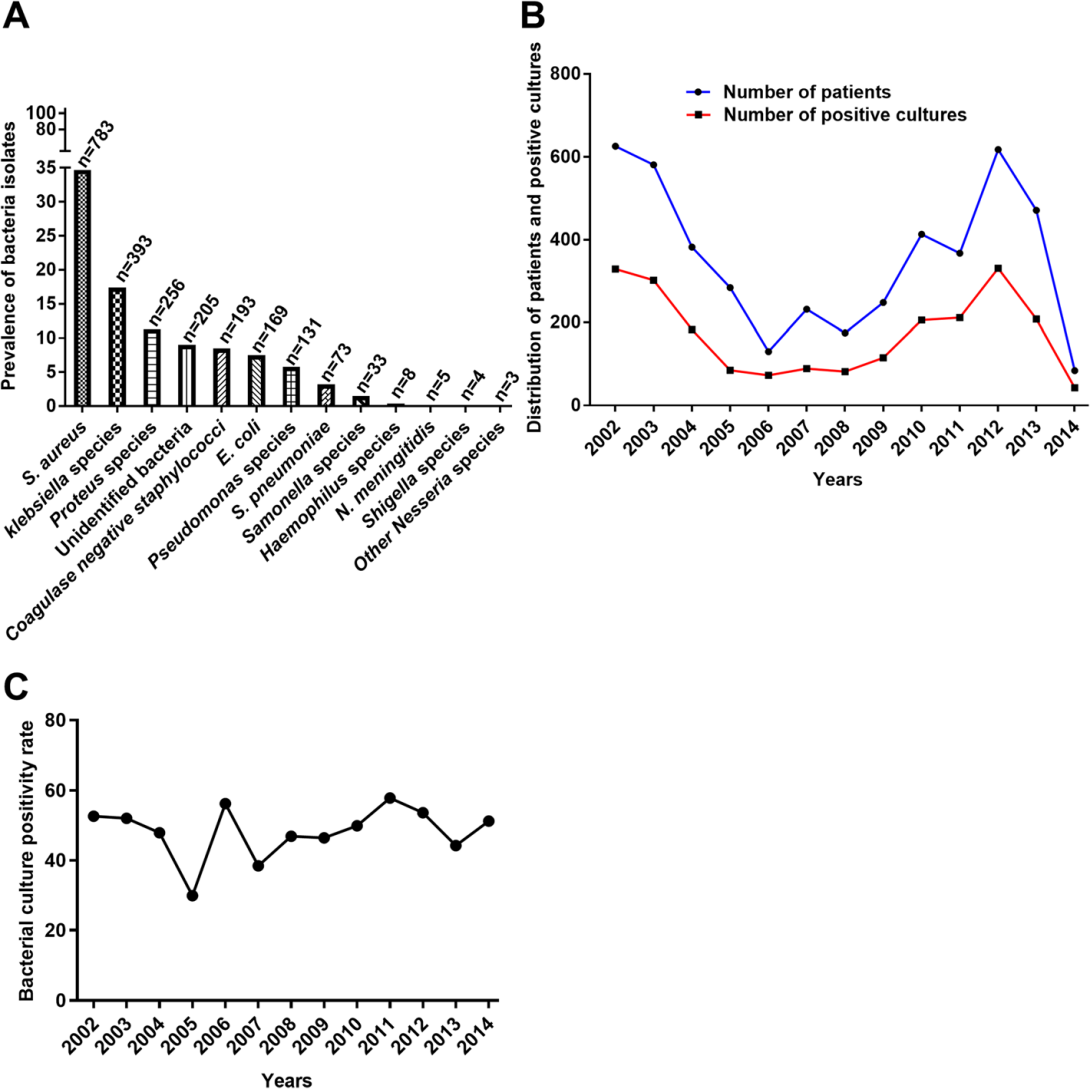
Prevalence of bacterial pathogens over the 13-year period. **a** Shows the prevalence of isolates during the study period. **b** Describes the distribution of patients alongside positive cultures throughout the study period. **c** Demonstrate the culture positivity rates in different years of the investigation period

To evaluate the prevalence of bacteria pathogens over the study period, we determined the overall numbers of individual isolates and established the comprehensive figure of pathogens per year. *Staphylococcus aureus* was the most commonly isolated bacterium (783, 34.7%) (Fig. 3a). Other common isolates included *Klebsiella* species (393, 17.4%), *Proteus* species (256, 11.4%), *Coagulase-Negative Staphylococcus* (193, 8.6%), *Escherichia coli* (169, 7.5%) and *Pseudomonas* species (131, 5.8%; Fig. 3a). The pattern of number of patients over the years matched the pattern of positive cultures. As the number of patients increased or decreased so did the number of positive cultures (Fig. 3b). Altogether, the culture positivity rate was approximately 48.9% (2258/4611). Assuming that each patient submitted one sample, the highest culture positivity rate was recorded in 2011 (212/367; 57.7%) whereas the lowest rate was registered in 2005 (85/284; 29.9%; Fig. 3c). In 2011, 54.2% (115/212) of the bacteria isolates were gram negative with *Klebsiella* species being dominant (25/115; 21.7%).

### Distribution of bacterial isolates according to demographics

**Fig. 4:**
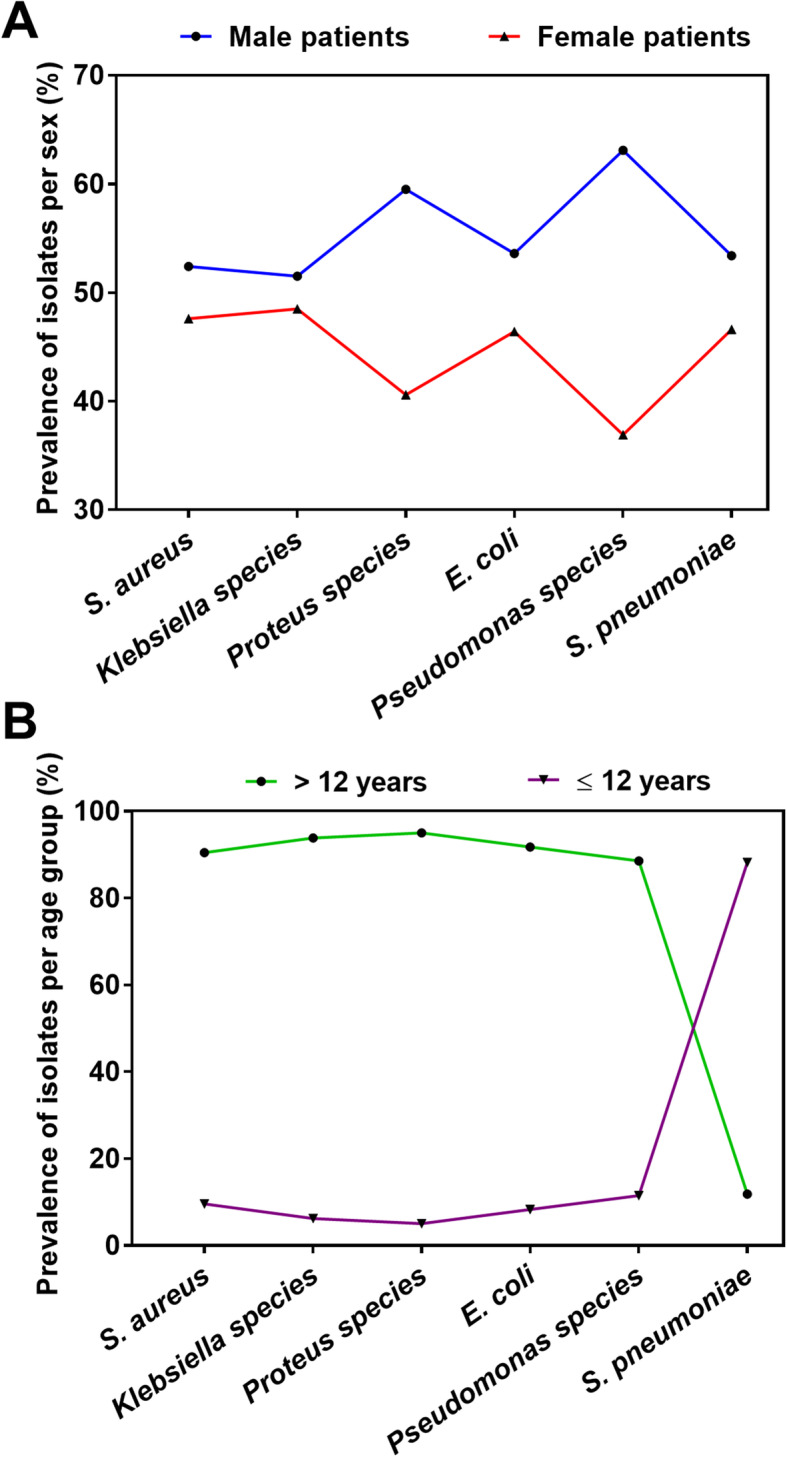
Prevalence of bacterial isolates per gender and age group. Isolated bacteria were distributed according to sex (**a**) and age group (**b**) of the patients

To identify the most susceptible group from bacterial infections, isolates were distributed according to gender and age groups of the patients. The percentage of male and female patients whose samples were positive for bacterial culture was roughly the same i.e. 51 and 49% respectively (Fig. 4a). A significant number of bacterial isolates were isolated from patients aged more than 12 years (Fig. 4b). Largely, the distribution of isolates among male and females was fairly similar with prevalence rates of within 50% except for *Pseudomonas* species which were isolated more in males than females (63.1, 36.9% respectively; Fig. 4a). Most of the isolates were recovered from patients older than 12 years except *S. pneumoniae* which was isolated predominantly in clients younger than 12 years (88.2%; Fig. 4b).

### Antimicrobial susceptibility profiles of major bacterial isolates

To assess antimicrobial susceptibility, most commonly isolated bacteria were tested for their susceptibility to the most commonly used antimicrobials at the hospital during the 13-year period. In comparison to other pathogens, *S. pneumoniae* demonstrated the highest rate of susceptibility to most of the antibiotics except for gentamicin where 40% of the strains showed resistance (Table 2). In this study, ciprofloxacin followed by gentamicin and cotrimoxazole proved to be the most effective antimicrobials with overall pathogen susceptibility rates of 84, 75 and 70% respectively (Table 2). Nonetheless, worrying levels of antimicrobial resistance was registered in the study. On the whole, *Pseudomonas* species exhibited relatively the highest resistance to most antibiotics. Overall, high rates of antimicrobial resistance to erythromycin (49%), chloramphenicol (47%), and nalidixic acid (42%) were recorded (Table 1).

**Table 2:**
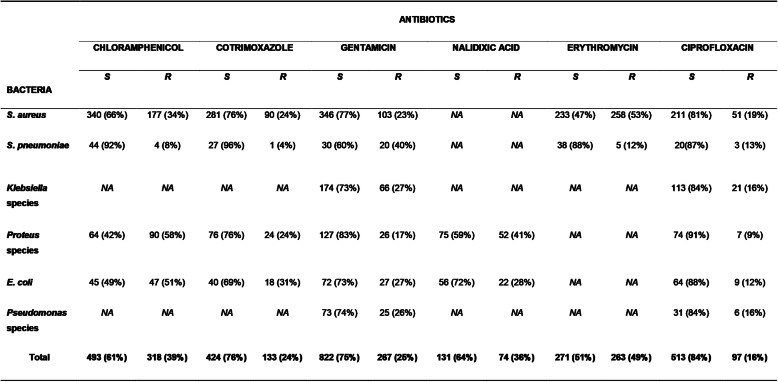
Antimicrobial susceptibility patterns of major bacterial pathogens against commonly used antimicrobials during 2002–2014 period. The numbers in the table represent isolates found to be susceptible and resistant to the antimicrobials

**Key**: ***NA*** Not Applicable, ***S*** Susceptible, ***R*** Resistance

## Discussion

Understanding distribution of microbial pathogens and their associated infections is essential for controlling infectious diseases and monitoring of antimicrobial resistance. The current study aimed at establishing the prevalence of common pathogenic bacteria including their antimicrobial susceptibility patterns and distribution according to specimens, age groups and sex at Mzuzu Central hospital. We report a high prevalence of bacteria isolates with variability in susceptibility to key antimicrobials used during the study period. Most isolates displayed high resistance to erythromycin, gentamicin, chloramphenicol, nalidixic acid and cotrimoxazole. Conversely, majority of the isolates were sensitive to ciprofloxacin.

While a significant number of similar studies in Malawi were limited to investigating blood stream bacterial infections [5, 13–18], our study demonstrated the spread of bacterial infections where other body sites were surveyed (Fig. 2a-b). The high prevalence of bacteria isolates observed in this study (Fig. 3a) highlights the need for effective monitoring and surveillance of bacterial infections in resource-limited health care settings. There was a sharp decrease in number of isolates tested between 2003 and 2006 (Fig. 3b), followed by a slow appreciation in subsequent years. Among other reasons, we suggest that this might have been as a result of reduced capacity of the laboratory to perform microbiology testing services. In 2004, the Malawi government had parted ways with a major donor supporting services at Mzuzu Central Hospital. Several services including laboratory services were negatively affected until a few years later when the government devised ways to fill the gap.

Similar to previous investigations [19–24], our findings revealed that *S. aureus* was the predominant cause of bacterial infections (Fig. 3a). *S. aureus* is the common cause of skin and soft tissue infections [25, 26]. Hence it is not surprising that we observed considerably high number of isolates from OPD, male surgical ward and female surgical ward following culture of pus obtained from wound and surgical site infections. The subsequent prevalent pathogens in the study were *Klebsiella* and *Proteus* species (Fig. 3a), which were also mostly recovered from wound pus. Likewise, this observation is comparable to investigations conducted in other countries [22, 23, 26–28].

The fluctuations in the number of patients that were provided with microbiology services correspond with the number of positive cultures. As the number of patients increased, positive cultures also increased and vice versa (Fig. 3b). The relatively high bacterial culture positivity rates observed (Fig. 3c) in this study could suggest good culture and bacterial isolation techniques. On the other hand, this could also be as a result of more contaminants being isolated, as most of the isolates i.e. *S. aureus* were isolated from pus, which can easily be contaminated with skin flora.

Except for *S. pneumoniae* most of the pathogens were isolated from patients > 12 years old and adult patients (Fig. 4b). The study registered more adults than children hence the observation that more isolates were recovered from adults corresponds with the high number of adult clients registered. *S. pneumoniae* is a common cause of bacterial meningitis in children [29], as such, our finding is consistent with literature. Correspondingly, our study showed that *S. pneumoniae* was predominantly isolated from CSF largely collected from children.

In general, all major isolates showed relatively high resistance to essential antimicrobials used during the study period (Table 2). Similar to previous studies [19, 30], our investigation showed higher rates of *S. aureus* resistance to chloramphenicol and erythromycin. Methicillin-resistant *Staphylococcus aureus* (MRSA) has significantly contributed to antimicrobial resistance globally [31], hence the drawback in this investigation is that *S. aureus* isolates were not tested to determine if some of the isolates were of MRSA origin.

*S. pneumonia* is a significant cause of pneumonia, sepsis, bacterial meningitis and death in Malawian children and adults [32–35]. Chloramphenicol is one of the antibiotics used in the treatment of bacterial meningitis including invasive pneumococcal diseases in Malawi. Previous investigations in Malawi reported higher rates of *S. pneumoniae* resistance to chloramphenicol which is in contrast to the findings in our study [5, 19]. Our observation needs to be interpreted with care since the number of isolates analysed in the current study is smaller than previous studies, hence, comparing the rates of antimicrobials resistance may not give a true reflection of the real situation. However, in general all major isolates exhibited high resistance to chloramphenicol.

The high rates of resistance to chloramphenicol, cotrimoxazole and nalidixic acid by major gram-negative organisms in the study is of concern as we observed a large population of patients affected during the study period. This is of importance as studies have shown an increased risk of subsequent infection and mortality after hospital discharge following colonisation with drug resistant gram-negative bacteria [36]. Whilst drug resistant Gram-negative bacteria are recognised as a global problem in resource limited countries, the threat is much higher in areas with poor healthcare infrastructure and poor surveillance for antimicrobial resistance.

According to the Malawi Standard Treatment Guidelines, the antimicrobials used in the study are essential in the treatment of several conditions including sepsis, chronic diarrhoea and infant bacillary dysentery respectively [37]. In addition, cotrimoxazole is used as a prophylactic treatment for bacterial infections in HIV positive clients in the WHO clinical stages II, III and IV [37]. Overall, it is encouraging that ciprofloxacin proved to be relatively effective against most pathogens. In Malawi, ciprofloxacin is used in the treatment of bacillary dysentery in adults, sepsis, and also as a prophylactic treatment for meningococcal meningitis in adults [37].

### Limitations

Some of the limitations of this study include lack of patient history on previous antimicrobial use. Consequently, this might have an influence on the observed rate of antimicrobial resistance. Secondly, due to limited availability of raw data, we were not able to capture an in-depth analysis of critical epidemiological data such as name of referral hospitals for the patients, onset of disease, occupation, and HIV status. The study was conducted at one tertiary hospital in northern Malawi as such the findings cannot be generalized to the whole country. Lastly, laboratory data on pathogen isolation, identification and susceptibility testing was generated using conventional methods, hence the results may be limited. Nevertheless, this study has provided an awareness on common microorganism isolated in our set up, their distributions and antimicrobial resistance pattern. This data could help practitioners and policy makers to make informed decisions on management of patients.

## Conclusions

As a result of several social and public health interventions, Malawi has over the years recorded a marked decline in the incidence of bacterial bloodstream infections [5]. However, this study has demonstrated an increase in bacteria burden in sites other than blood stream as well as a concurrent increase in prevalence of antimicrobial resistance. The increased rates of antimicrobial resistance revealed in the study could be due to poor clinical practices or irrational use of antibiotics by the public. Nevertheless, to fight against antimicrobial resistance, a local epidemiological surveillance program is needed to guide in the generation of evidence-based guidelines for the treatment and management of bacterial infections.

## Data Availability

All data related to this study can be accessed at Mzuzu Central hospital upon consent is given from the authorities.

## Abbreviations

AMR: Antimicrobial Resistance
BAP: Blood Agar Plate
CAP: Chocolate Agar Plate
CSF: Cerebral Spinal Fluid
ESBL: Extended Spectrum beta-lactamase
MAP: MacConkey Agar Plate
MCH: Mzuzu Central Hospital
MRSA: Methicillin Resistant *Staphylococcus aureus*
NHSRC: National Health Science Research Committee
OPD: Out Patient Department
sSA: Sub-Saharan Africa
UKNEQAS: UK National External Quality Assessment System
ZIMQAP: Zimbabwe Quality Assurance Program
